# Activation of a chondrocyte volume-sensitive Cl^−^ conductance prior to macroscopic cartilage lesion formation in the rabbit knee anterior cruciate ligament transection osteoarthritis model

**DOI:** 10.1016/j.joca.2016.05.019

**Published:** 2016-10

**Authors:** K. Kumagai, F. Toyoda, C.A. Staunton, T. Maeda, N. Okumura, H. Matsuura, Y. Matsusue, S. Imai, R. Barrett-Jolley

**Affiliations:** † Department of Musculoskeletal Biology, Institute of Aging and Chronic Disease, University of Liverpool, UK; ‡ Department of Orthopedic Surgery, Shiga University of Medical Science, Japan; § Department of Physiology, Shiga University of Medical Science, Japan

**Keywords:** Osteoarthritis, Cartilage, Ion channels, ACLT, Chloride channel, Anion channel, Caspase, Apoptosis

## Abstract

**Objective:**

The anterior cruciate ligament transection (ACLT) rabbit osteoarthritis (OA) model confers permanent knee instability and induces joint degeneration. The degeneration process is complex, but includes chondrocyte apoptosis and OA-like loss of cartilage integrity. Previously, we reported that activation of a volume-sensitive Cl^−^ current (*I*_Cl,vol_) can mediate cell shrinkage and apoptosis in rabbit articular chondrocytes. Our objective was therefore to investigate whether *I*_Cl,vol_ was activated in the early stages of the rabbit ACLT OA model.

**Design:**

Adult Rabbits underwent unilateral ACLT and contralateral arthrotomy (sham) surgery. Rabbits were euthanized at 2 or 4 weeks. Samples were analyzed histologically and with assays of cell volume, apoptosis and electrophysiological characterization of *I*_Cl,vol_.

**Results:**

At 2 and 4 weeks post ACLT cartilage appeared histologically normal, nevertheless cell swelling and caspase 3/7 activity were both significantly increased compared to sham controls. In cell-volume experiments, exposure of chondrocytes to hypotonic solution led to a greater increase in cell size in ACLT compared to controls. Caspase-3/7 activity, an indicator of apoptosis, was elevated in both ACLT 2wk and 4wk. Whole-cell currents were recorded with patch clamp of chondrocytes in iso-osmotic and hypo-osmotic external solutions under conditions where Na^+^, K^+^ and Ca^2+^ currents were minimized. ACLT treatment resulted in a large increase in hypotonic-activated chloride conductance.

**Conclusion:**

Changes in chondrocyte ion channels take place prior to the onset of apparent cartilage loss in the ACLT rabbit model of OA. Further studies are needed to investigate if pharmacological inhibition of *I*_Cl,vol_ decreases progression of OA in animal models.

## Introduction

Chondrocyte apoptosis is an important contributor to the development and growth of healthy articular cartilage^1^. In the process of normal bone growth and endochondral ossification, terminally differentiated chondrocytes are removed from the calcified cartilage by apoptosis prior to the transition to bone^1^. There is also evidence that an increased incidence of chondrocyte apoptosis during aging is responsible for the hypocellularity associated with degradation and/or pathological remodeling of the cartilage matrix, and exacerbates the risk of degenerative joint diseases such as osteoarthritis (OA)^1^. Study of this in biopsies from OA patients can suffer from the limitation that OA is typically presented at an advanced stage. To address this issue, this study used a knee instability anterior cruciate ligament tear rabbit model (ACLT) which induces OA-like degradative changes in the joint^2^, ^3^ and investigated changes in chondrocyte physiology taking place prior to apparent macroscopic changes of the cartilage.

During onset of OA, disruption of the collagen network of cartilage is also accompanied by an increase in water content and a corresponding decrease in cartilage osmolality^4^, ^5^. Decrease in extracellular osmolality causes chondrocytes to swell^6^, and cell swelling is, in general, a trigger for apoptosis^7^. Articular chondrocytes are also exposed to perturbation of osmotic pressure and ionic composition during normal use^6^. Chondrocytes have mechanisms in place to oppose cell swelling (referred to as regulatory volume decrease, RVD) and several ion channels and transporters have been implicated in this cell-volume regulatory process (for reviews see^6^, ^8^ and these have been implicated in pathogenesis of OA.

In previous studies, we and others have shown that volume-sensitive Cl^–^ channels (*I*_Cl,vol_) are functionally expressed by articular chondrocytes and involved RVD^9^, ^10^, ^11^. Aberrant activation of *I*_Cl,vol_ under iso-osmotic conditions contributes to the cell-shrinkage associated with induction of apoptosis in cells including chondrocytes^12^, ^13^. We therefore hypothesized that the chondrocyte *I*_Cl,vol_ may also be associated with OA.

In the current report we measured *early* changes in chondrocyte volume regulation and of the volume-sensitive Cl^−^ current (*I*_Cl,vol_), together with an apoptotic marker (caspase activity) and cartilage histology. Our results show that *I*_Cl,vol_, cell-volume control and caspase activity are all altered following ACLT, but prior to the manifestation of histologically detectable OA.

## Methods

Brief methods are included below, with more detailed methods in the “Supplementary methods” document. All experimental protocols conformed to The Guide for the Care and Use of Laboratory Animals (National Research Council 2011) and were approved by the Animal Care and Use Committee of Shiga University of Medical Sciences. All experiments used adult male white rabbits (body weight, 2.5–3 kg). Sham and ACLT surgical induction were conducted on right knees.

### Histological examination

3 μm thick sections were obtained from the femoral side of patellofemoral joints and stained with toluidine blue and safranin-O as for proteoglycans and glycosaminoglycans. For overall evaluation of the cartilage area, tissues were graded by three blinded observers using both the Mankin score system^14^ and OARSI histopathology score^15^.

### Isolation of rabbit articular chondrocytes

Articular chondrocytes were isolated using an enzymatic dissociation procedure similar to that described previously^16^ with modifications^10^. Dispersed chondrocytes were washed three times, re-suspended in DMEM supplemented 40 mM mannitol (∼360 mosmol/L) and used within 8 h.

### Caspase-3/7 activity measurement

Caspase-3/7 activity was measured as an indicator of apoptosis using the Caspase-Glo 3/7 assay system (Promega, Madison, WI, USA). The luminescent signal was measured with a luminometer (Infinite M200, Tecan, Männedorf, Switzerland).

### Cell swell assay

Cell size measurements and patch-clamp experiments were conducted on round-shaped chondrocytes. Live chondrocytes microscopy images were captured (at 1 min intervals) before and during a hypo-osmotic challenge at a 2560 × 1920 pixel resolution using a CCD digital camera (DS-Fi1, Nikon) equipped with a DS-L2 control unit (Nikon). The cell cross-sectional areas were calculated using Image-J (NIH, Bethesda, MD, USA). These were each normalized to their respective initial iso-osmotic size.

### Electrophysiology

Whole-cell membrane currents were recorded from isolated chondrocytes. Square-step and voltage-ramp protocols were used to record whole-cell currents. Hypotonic-isotonic difference currents were calculated by subtracting individual currents under isotonic conditions, from the equivalents in hypotonic conditions. We calculated conductance plots using Boltzmann transformations of data to separate the underlying whole-cell ion conductance from the Ohmic driving force for ion flow. These data were then fit by Boltzmann equations^17^ as follows:

(1) $$g_{\left(Vm\right)}=\frac{g}{\left(1+\exp\left(V_{m}-V_{h}\right)/k\right)}$$

where *g* is the maximal conductance, *V*_*h*_ is the *Vm* at which the conductance is half-activated (“midpoint”) and *k* is the slope of activation.

### Statistical analysis

Data are written as means (95% confidence intervals), with the number of animals (cell isolations) and cells from which measurements were made indicated by *N* and *n*, respectively. Statistical comparisons were made using either Student's *t*-test or a general linear model ANOVA (Minitab version 17, Minitab Ltd., Coventry UK) as stated.

### Solutions and chemicals

The iso-osmotic external solution used for the patch-clamp experiments contained (in mM): mannitol 150, NaCl 100, MgCl_2_ 2.0, BaCl_2_ 2.0, GdCl_3_ 0.03, glucose 5.5, and Hepes 10 (pH 7.4). Measured osmolality was approximately 360 mOsm. This osmolality was chosen because it resembles that of native cartilage. The pipette solution contained (in mM): caesium aspartate 135, CsCl 30, TEA-chloride 20, MgCl_2_ 2.0, Tris-ATP 5.0, Na_2_-GTP 0.1, EGTA 5.0, and Hepes 5.0 (pH 7.2). The iso-osmotic external solution used for measuring cell swell contained (in mM): mannitol 180, NaCl 90, KCl 5.4, CaCl_2_ 1.8, MgCl_2_ 0.5, NaH_2_PO_4_ 0.33, glucose 5.5, and Hepes 5.0 (pH 7.4). The hypo-osmotic external solution was made by omitting mannitol.

## Results

### Histological observation

**Fig. 1:**
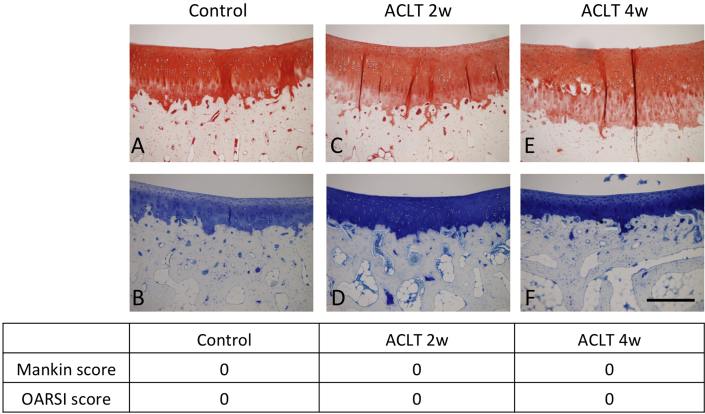
**Cartilage histology following ACLT**. Histological findings after Safranin O (A, C, E) and toluidine blue (B, D, F) staining in the three groups (sham (control) (A, B), ACLT 2w (C, D) and ACLT 4w (E, F)). Scale bar: 2 mm. All areas of ACLT 2-week and 4-week were preserved with normal appearance. Mankin score and OARSI score were also normal.

2 and 4 weeks following surgical treatment, tissue slices were prepared and assessed according to both the Mankin and OARSI osteoarthritis scoring systems^14^, ^15^. All areas of the 2 and 4-wk specimens of the ACLT knees were found to have macroscopically normal histological appearance in both toluidine blue and safranin-O staining (Fig. 1).

### Cell-volume control assay

**Fig. 2:**
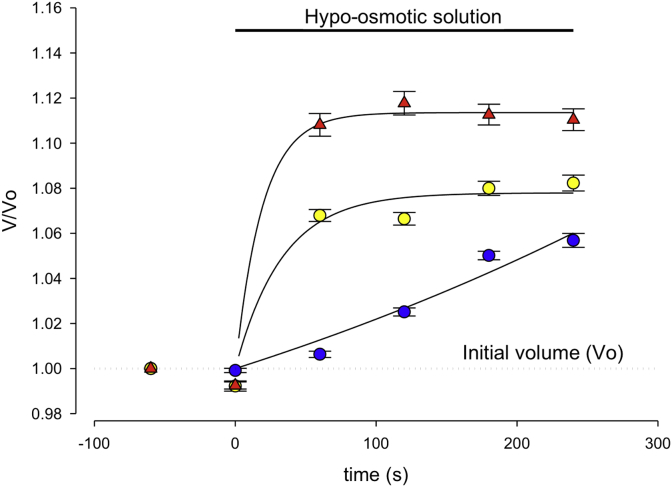
**Control and ACLT Chondrocyte Swell Data**. Changes in cell (chondrocyte) cross-sectional area in cells from sham (blue circles), and ACLT after 2 weeks (ACLT 2W, yellow circles) and 4 weeks (ACLT 4W, red triangles). Isotonic (360 mOsm) solution was changed to hypotonic solution (180 mOsm) at time 0s. Exposure of chondrocytes to hyposmotic solution led to a rapid increase in relative cell size (6% swell; i.e., 1.06, 95% CI 1.021–1.099 at 5 min, *n* = 15, *N* = 5) in control cells, and this swell was significantly greater with cells from ACLT 2W and 4W tissue (ACLT 2wk; 1.08, 95% CI 1.060–1.020 at 5 min, *n* = 21, *N* = 5, *P* ≤ 0.0005: ACLT 4wk; 1.11, 95% CI 1.071–1.149 at 5 min, *n* = 11, *N* = 5, *P* ≤ 0.0005. There was no significant change in absolute initial volumes (control 875, 95% CI 848–902 μm; ACLT 2wk 726, 95% CI 707–745 μm and ACLT 4wk 935, 95% CI 915–955 μm). All *P-values* were obtained from Dunnett's *post-hoc* multiple comparison against control).

To analyze whether changes in cellular phenotype had developed in chondrocytes from the macroscopically normal cartilage, we began by assessing chondrocyte cell-volume properties. We measured relative cell-volume response from isolated sham (control), ACLT 2wk and ACLT 4wk rabbit cartilage isolated chondrocytes, using our cell-swell assay. Cells were incubated in 360 mOsm solution and then exposed to a (180 mOsm) hypo-osmotic external solution. The degree of hypo-osmotic cell swelling was evaluated by measuring the cross-sectional area of cell images. As shown in Fig. 2, exposure of a chondrocyte to hypo-osmotic solution led to a rapid increase in relative cell size (approx. 6% swell, Fig. 2) compared to that in control cells. Figure 2 also shows the time-course of cell swell under these conditions. Chondrocytes from ACLT 2wk and 4wk rabbit cartilage tissue showed a more rapid increase in cell size than controls, both reaching a highly significantly greater relative volume at 5 min post hypotonic challenge (approx. 8% and 11% swell respectively, Fig. 2). These changes could result from a reduced capacity of chondrocytes to regulate their cellular-volume at a very early stage of OA progression.

### ACLT induced caspase 3/7 activity

Changes in cellular volume regulation are frequently linked to progression of apoptosis. To investigate this we used an apoptosis assay. We used a caspase-3/7 activity assay since it has been previously demonstrated that this is a major step in apoptotic cell death^18^.

**Fig. 3:**
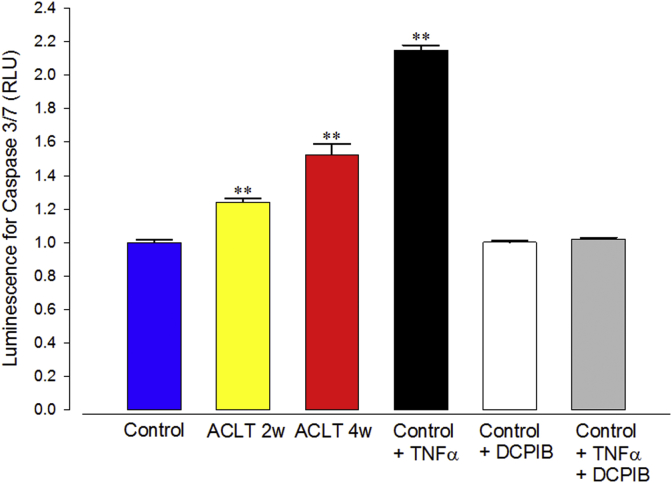
**Caspase-3/7 activity, measured from chondrocytes**. Caspase activity is given in terms of luminescent intensity (arbitrary units) measured with the Caspase-glo 3/7 assay system described in the methods. Columns represent cells from control tissue, ACLT 2 and 4w represent that from tissue 2 and 4 weeks after ACLT. Control + TNFα is a positive control where control rabbit chondrocytes were exposed to 10 ng/ml TNFα for 24 h, Control + DCPIB is a negative control (cells exposed to 20 μM DCPIB). Control + TNFα + DCPIB combines the TNFα and DCPIB treatments, as described for the previous columns. Asterisks represent data significantly increased from control (Minitab GLM ANOVA, Dunnett's multiple comparison *post hoc* test **P ≤ 0.0005). Caspase-3/7 activity was markedly elevated in chondrocytes from ACLT 2w (1.24, 95% CI 1.20–1.28, *n* = 20, *N* = 5, *P* ≤ 0.0005), 4w (1.52, 95% CI 1.40–1.64, *n* = 20, *N* = 5, *P* ≤ 0.0005) animals and Control + TNFα (2.15, 95% CI 2.09–2.21, *n* = 20, *N* = 5, *p* ≤ 0.0005). There was no significant increase from control with either DCPIB alone, or TNFα +DCPIB). Note, the increase between 2wk and 4wk was also statistically significant *P* = 0.0026. Each cell numbers 1 × 10^5^ cells/ml.

As a positive control, we used TNFα treatment (24 h 10 ng/ml). We also investigated if this process could be prevented by an inhibitor of the swelling/volume sensitive chloride channel blocker DCPIB. TNFα induced a significant increase in caspase-3/7 activity and this was abolished by DCPIB. DCPIB alone had no effect on population incidence of apoptosis. We also determined incidence of apoptosis in ACLT cartilage and the controls. We found caspase-3/7 activity to be significantly elevated in both 2wk and 4wk ACLT OA models (Fig. 3) in comparison with control.

### Constitutive anion channel activity in ACLT chondrocytes and controls

**Fig. 4:**
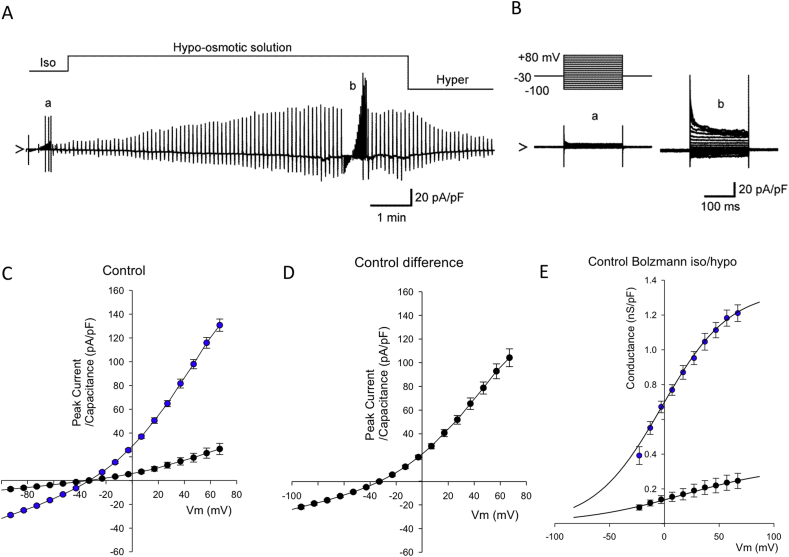
**Control chondrocyte whole-cell data**. Switch from isotonic solution to hypotonic solution (as described in the methods) resulted in increased whole-cell conductance in chondrocytes from sham control rabbit tissue. Solutions optimized for detection of chloride conductances (see Methods). (A) Representative continuous raw current data trace during switch from isotonic to hypo and then hypertonic solution. Constant ramps (dV/dt 0.25 V/s) were applied at 6s intervals, with full current voltage protocols run at points indicated by “a” and “c” (see B–E). (B) Expanded view of the raw current voltage data shown in (Aa,b). The upper panel illustrates the voltage protocol; the lower traces “a” and “b” are the resultant current traces. Superimposed current traces are in response to 200 ms square-steps applied from a holding potential of −30 mV to test potentials of +80 through −100 mV in 10 mV steps. (C) Mean current voltage data from a number of protocols such as that shown in A and B. The filled black circles indicate recordings in isotonic solution. There is a large activation of current in the presence of hypotonic solution (filled blue circles). (D) Subtraction of the current in isotonic from the current in hypotonic solution gives the hypotonic activated (difference) current. Current reverses near to the calculated reversal potential for chloride ions (*E*_Cl_^−^ 19 mV). (E) Boltzmann transformation of the current–voltage curves in C (junction potential corrected data). Control isotonic data (black filled circles) is fit with midpoint (*V*_*h*_) 40 (95% CI 16.5–63.5) mV, slope (*k*) 62 (95% CI 42.4–81.6) mV and maximum conductance (*g*) of 364 (95% CI 268–460) pS/pF. In hypotonic solution (blue filled circles), the mean control chondrocyte Boltzmann curve is significantly; shifted to the left (*V*_*h*_ = −2, 95% CI −11.8 to 7.8 mV, *P* ≤ 0.0005), steeper slope (*k* = 31, 95% CI 23.2–38.8 mV *P* = 0.018 and larger (maximum conductance *g* = 1350, 95% CI 1250–1450 pS/pF *P* = 0.009). Note that these are Benjamini–Hochberg adjusted *P-values*.

Articular chondrocytes express a wide variety of cation and anion channels important for cell volume control^8^. We recently showed a major contribution of *I*_Cl.*vol*_ to drug doxorubicin-induced chondrocyte apoptosis in chondrocytes^13^ and so the current study focused on anion currents. Isolated rabbit articular chondrocytes were investigated under conditions designed to minimize Na^+^ currents (a holding potential of −30 mV), Ca^2+^ currents (removal of Ca^2+^ from the external solution), K^+^ currents (omission of K^+^ from the internal solution and addition of BaCl_2_ to the external solution), and electrogenic Na^+^/K^+^ pump current (omission of Na^+^ and K^+^ from internal and external solutions, respectively). Gd^3+^-sensitive stretch-activated channels^19^ were also blocked by adding 30 μM GdCl_3_ to the bath. Gd^3+^ also blocks the majority of TRP cation channels^20^. Figure 4A and B shows representative whole-cell currents under these conditions, in response to 200 ms square voltage-clamp steps applied from a holding potential of −30 mV to test potentials of +80 to −100 mV in 10 mV steps. In our previous pharmacological studies, we established this was a chloride conductance^9^, ^10^. To allow in depth analysis of the whole-cell conductance we transformed the current–voltage data to whole-cell conductance plots [Fig. 4(E)]. These were then fit with Boltzmann curves (Equation (1)) showing that maximum conductance (*g*) was significantly increased by ACLT surgery, but that slope (*k*) and half-maximum conductance activation (*V*_*h*_) were not significantly changed [Fig. 4(E)].

### Hypotonicity-activated anion channel activity in ACLT chondrocytes and controls

**Fig. 5:**
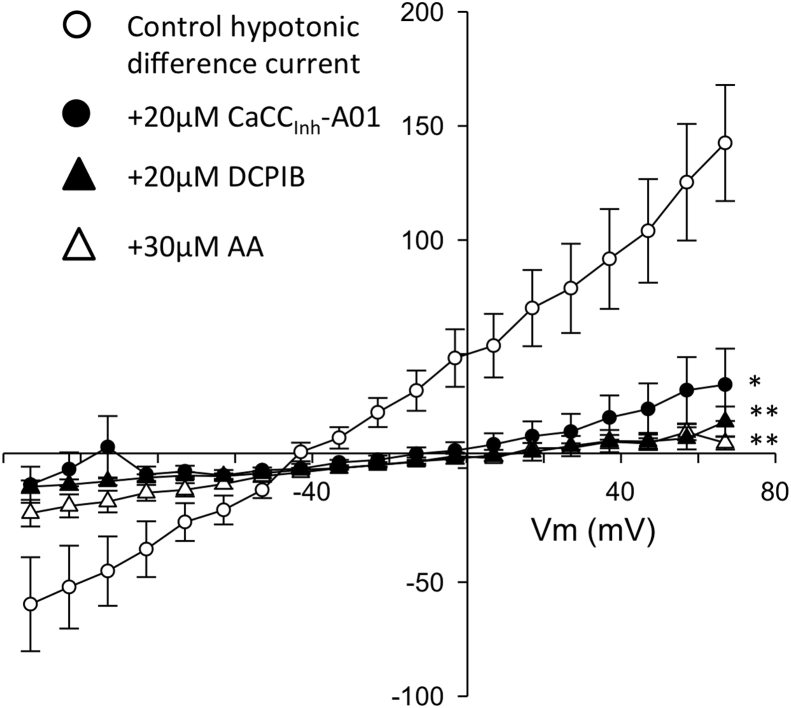
**Pharmacological analysis of the hypotonically activated conductance**. Hypotonic difference currents, recorded and calculated as in Fig. 4(D). Empty circles are under control conditions, solid circles in the presence of 20 μM CaCCinh-A01, solid triangles in the presence of 20 μM DCPIB and empty circles in the presence of 30 μM AA. *P = 0.007, **P < 0.0005 general linear model ANOVA (Vm * drug) with Tukey's Pairwise multiple comparisons (post-hoc) test. This pairwise comparison test also revealed that the difference curves for DCPIB and AA were significantly more inhibited than that in the presence of CaCCinh-A01 (*P*-values = 0.012 and 0.020 respectively). DCPIB and AA curves were not themselves significantly different.

**Fig. 6:**
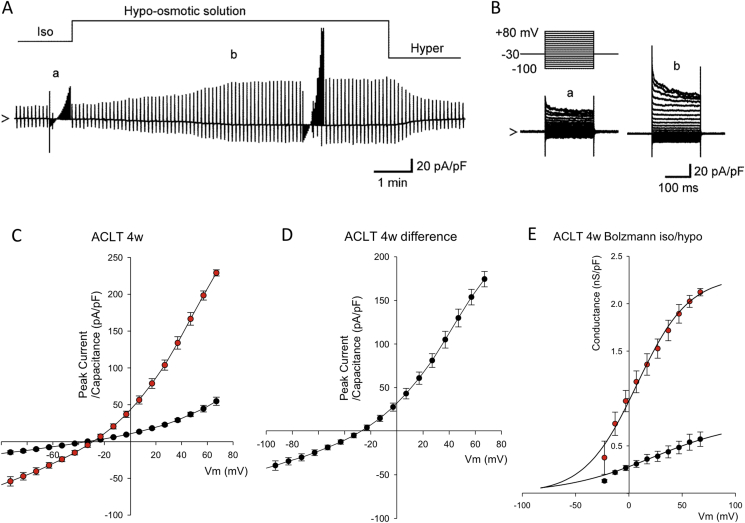
**Whole-cell data from ACLT 4wk chondrocytes**. (A) Raw data from the same protocol as that described in Fig. 4, but using chondrocytes from 4W ACLT animals. Switching from isotonic solutions to hypotonic solutions activates a large voltage-gated current, with full current voltage protocols (see B–E) run at points indicated by “a” and “b”. (B) Expanded view of the raw current voltage data shown in (Aa,b). The upper panel illustrates the voltage protocol; the lower traces “a” and “b” are the resultant current traces. (C) Mean current voltage data from a number of protocols such as that shown in A and B. Currents recorded in isotonic solution are shown as black filled circles. There is a large activation of current in the presence of hypotonic solution (red filled circles). (D) Subtraction of the current in isotonic from the current in hypotonic solution gives the hypotonic activated (difference) current. As Figure 4, this current reverses near to the calculated *E*_Cl_^−^. (E) Boltzmann transformation of the current–voltage curves in C. 4wk ACLT isotonic data (black filled circles) is fit with midpoint (*V*_*h*_) 23 95% CI (−0.5 to 46.5) mV, slope 42 (95% CI 34–50) mV. These are not significantly different to the control isotonic equivalent values (*P* = 0.35 and *P* = 0.11 respectively). Isotonic maximum conductance (762, 95% CI 58–966 pS/pF) was significantly greater than that of control chondrocytes (*P* = 0.004). In hypotonic solution (red filled circles), the mean 4W ACLT chondrocyte Boltzmann curve was not significantly shifted (*V*_*h*_ 10, 95% CI 2.2–17.8 mV) from that in isotonic *(P* = 0.35) or that or from the control hypotonic values (*P* = 0.22), but was significantly steeper slope (*k* 25, 95% CI 21.1–28.9 mV *P* = 0.014) and larger (maximum conductance 2290, 95% CI 2200–2380 pS/pF *P* ≤ 0.0005) than the ACLT isotonic conductance curves. The ACLT hypotonic conductance value was also greater than the control equivalent (*P* ≤ 0.0005), but not significantly steeper (*P* = 0.263). Note that these are Benjamini–Hochberg adjusted *P-values*. Direct comparison of difference current derived Boltzmann curves between Control and 4 W ACLT are shown in Fig. 6.

Exposure of the chondrocyte to hypo-osmotic solution caused increase in the whole-cell current in chondrocytes from both control and ACLT cartilage [Figs. 4(C), (D) and 6(C), (D)]. The hypo-osmotic swelling-activated current was also largely time-independent at potentials negative to +50 mV and outwardly rectifying with a reversal potential [Fig. 4(E)] close to the calculated *E*_Cl_^−^. To investigate the molecular identity of the hypotonically activated chloride conductance, we measured difference currents of control cells with and without the presence of three different chloride channel blockers; CaCC_Inh_-A01 (a selective blocker of the Ca^2+^-activated chloride channel and TMEM16A/anoctamin-1/ANO-1)^21^, DCPIB (an inhibitor of the volume sensitive anion channel^22^), VSAC/VRAC) and arachidonic acid (AA) (an inhibitor of VRAC^23^). All three of these caused a significant inhibition of conductance, CaCC_Inh_-A01 significantly more effective than DCPIB or arachidonic acid (Fig. 5).

**Fig. 7:**
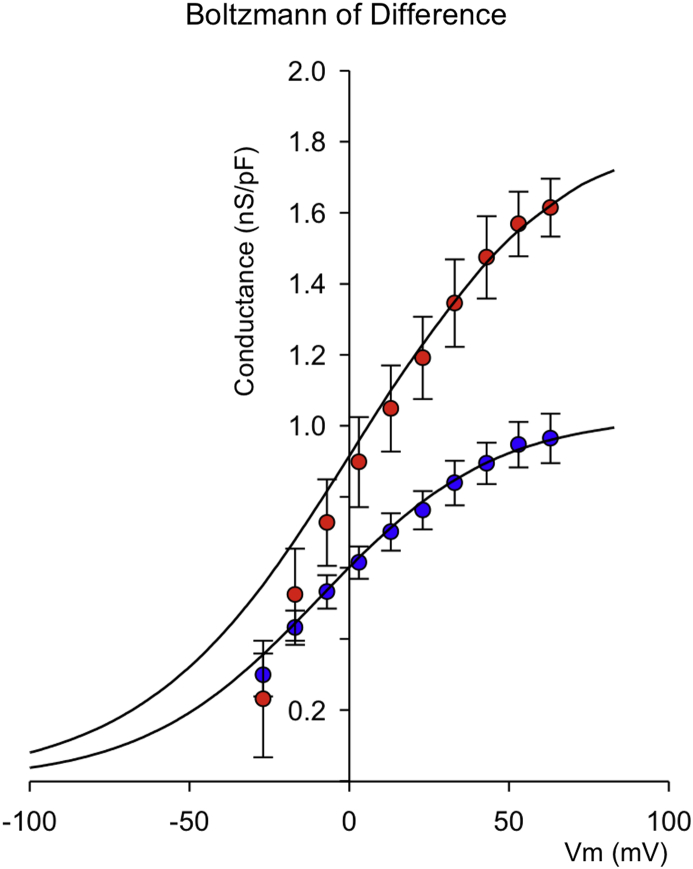
**Boltzmann analysis of control and 4W ACLT difference currents**: Boltzmann curves created from the Control (blue circles) and 4W ACLT (red circles) chondrocyte difference current data shown in Fig. 4, Fig. 5. The smooth lines are fits with the Boltzmann curves with parameters as follows: For control; *V*_*h*_ 68 (95% CI 58.2–77.8) mV, slope 31 (95% CI 23.16–58.84) mV, maximum conductance 1035 (95% CI 935–1135) pS/pF. For 4W ACLT curves the mean fitted parameters were: V_h_ 7 (95% CI −2.8 to 16.8) mV, slope 24 (20.8–27.92) mV, maximum conductance 2290 (2200–2380) pS/pF. Slope was not significantly different from control (*P* = 0.19), but both midpoint and maximum conductance were *P* ≤ 0.0005 for each). Note that these *P-values* are Benjamini–Hochberg adjusted.

To analyze ACLT induced changes in underlying conductance in detail, we Boltzmann transformed control and ACLT hypotonic difference currents and fitted these with Equation (1) [Fig. 6(E)]. In both controls and ACLT samples, hypotonicity results in a significant increase in maximum conductance and steeper slope (smaller absolute *k*). In control cells, the half maximum activation voltage (*V*_*h*_) of conductance was significantly shifted to the left (channels open at more negative potentials) by hypotonic challenge. This leftward shift of *Vh* was not apparent in ACLT cells. To analyze and quantify just the hypotonically activated current, without contamination of the tonically active current, we constructed difference currents, for currents in isotonic and hypotonic solutions. The hypo-osmotic swelling-activated difference current was also outwardly rectifying with a reversal potential [Fig. 4(E)] close to *E*_Cl_^−^. We Boltzmann transformed and fitted the hypotonic-isotonic difference currents in control and ACLT chondrocytes (Fig. 7). The difference current maximum conductance (*g*) was significantly greater in ACLT chondrocytes and shifted to the right compared to controls. The slope of activation was not significantly different.

## Discussion

In this study we used a rabbit joint destabilization model of early arthritis. We investigated samples prior to the development of macroscopic cartilage changes, but found that caspase-3/7 activity (apoptosis), cell-volume regulation and the chloride conductance *I*_Cl.vol_ were all significantly altered in ACLT treatment joints compared to sham controls.

The diagnosis of OA is mainly based on physical examination and radiograph (Kellgren–Lawrence grading (K-L Grading)) supported by laboratory tests such as C-reactive protein (CRP), erythrocyte sedimentation rate (ESR) or arthroscopy. Each of these diagnostic techniques has limitations: Radiographs provide positive results only after significant progression of disease^24^. CRP and ESR are indicators of inflammation, but are not site specific. Arthroscopy reveals damage to cartilage that is not visible on radiographs, but is an invasive technique. Magnetic resonance imaging is a useful alternative and non-invasive technique, but cost and availability can prevent routine use. Joint tissue degeneration is therefore usually advanced by the time the diagnosis and our research focus has shifted to understand the earliest physiological changes in cartilage or chondrocytes.

### Histological analysis of control and post ACLT samples

Our histological data convincingly show no significant difference in indicators of degeneration between any of the three groups using the Mankin score system^14^ or OARSI histopathology score^15^. These histological results are consistent with previous reports. For whilst a previous study did observe degenerative changes 4 weeks post ACLT, they were much less marked than longer term studies and there was no evidence of full-thickness ulceration. In addition, those changes that were observed were quite variable between samples^3^. Therefore, it appears that 4-week post-ACLT is, in rabbits, somewhat of a threshold stage of OA where macroscopic changes just begin to materialize.

### Changes in cell volume properties

Despite the lack of clear macroscopic histological changes in the 2 or 4-week ACLT cartilage samples, we did find a number of changes in chondrocyte cellular physiology. What triggers these changes prior to evident changes in matrix structure is unknown; possibilities include changes in mechanical loading of cartilage and thus resident chondrocytes^25^, or very early biochemical changes and induced stress, several of which have been shown to change early in models of OA^26^, ^27^. Potentially, even the established changes in cartilage osmolality^4^ could lead to secondary changes in chondrocyte phenotype. Since chondrocytes produce the enzymes that maintain cartilage, it seems logical that changes in chondrocyte phenotype could precede evident extracellular matrix changes. The first observation we made was that chondrocytes swelled more following ACLT treatment than sham controls. Since passive cell swell and RVD take place in parallel, this phenomenon could be due to a decreased capacity for RVD (which would otherwise oppose cell swell), a change in cytoskeletal properties, or an increase in cell membrane permeability to water which could arise from the increased aquaporin expression we previously reported to occur in some models of OA^28^. These hypotheses are not mutually exclusive, since it is likely that changes in any of the components of cell-volume regulation, such as aquaporins, *I*_*Cl*.*vol*_, BK etc^8^ could lead to an impaired ability to undergo RVD.

### Changes in apoptosis

One key consequence of altered cell-volume regulation is programmed cell death, apoptosis^29^, ^30^. Other important elements of the apoptotic pathway are the activity of a number of intracellular enzymes including the caspases^31^. We chose to investigate caspase activity, with a luminescence system optimized for detection of isoforms 3/7, since it is thought that all apoptosis pathways pass through these^31^. Our data showed that caspase-3/7 activity was increased at both 2-weeks and 4-weeks following ACLT confirming again, that cellular physiological changes had taken place prior to observable macroscopic degeneration.

### Electrophysiological changes

Several ion channels are involved with chondrogenesis^32^, chondrocyte migration^33^, volume control/apoptosis^34^, ^35^ and mechanotransduction^36^. Considerable interest has also surrounded the chondrocyte stretch activated BK channel^8^, ^37^, ^38^ and TRPV4 cation channels^39^, ^40^, ^41^, with TRPV4 being raised as a potential therapeutic target in a range of musculoskeletal diseases^42^. Anion channels are, however, equally important to cation channels and some of our own previous work has shown that a swell activated chloride channel *I*_Cl,vol_ is of particular importance to development of apoptosis in chondrocytes^13^. Our previous electrophysiological studies showed that *I*_Cl,vol_ (also referred to as the volume-sensitive organic osmolyte/anion) is functionally expressed in rabbit articular chondrocytes and is involved in cell volume maintenance mechanisms such as RVD^9^, ^10^. In addition to this physiological importance in the homeostatic regulation of cell volume, activation of *I*_Cl,vol_ has also been suggested to contribute to the cell shrinkage associated with apoptosis in several cell types^12^.

In the present study we measured whole-cell currents in conditions optimized for detection of chloride conductances (e.g., potassium free etc). We analyzed currents under both isotonic conditions and following hypotonic shock. Both “resting” (iso-osmotic) and hypotonic-activated currents were increase significantly in cells from joints 4 weeks post ACL. Detailed analysis reveals that hypotonic challenge of chondrocytes changed the maximum chloride conductance, the slope and midpoint for voltage activation of this conductance. Changes to all three of these parameters is fully consistent with the idea that hypotonicity activates a chloride conductance that is not present under isotonic conditions in control chondrocytes (i.e., *I*_Cl,vol_ see Fig. 4). Conversely, ACLT treatment significantly increased only the maximal conductance of chondrocytes without significantly changing either slope or midpoint of its activation; consistent with the hypothesis that there has been an increase in the number of active *I*_Cl,vol_ channels, rather than a change in their electrophysiological properties or expression of an entirely new/different channel. It is also consistent with a hypothesis that there has been an activation of *I*_Cl,vol_, even under isotonic conditions. Calculation of Boltzmann parameters from hypotonic-isotonic difference currents removes any contribution from constitutively active background conductances active under isotonic conditions.

Previously, two clearly distinguishable phenotypes of anion channels have been linked to cell apoptosis and cell volume regulation; the volume-regulated anion channel (VRAC) and the calcium-activated chloride conductance (CaCC)^23^, ^43^. Molecular identities have proven elusive with several gene products having been proposed; CLCN3 (CLC3), bestrophin, ANO-1 (an anoctamin) and the LRRC8 family^43^, ^44^. Latest evidence suggests that ANO-1 is synonymous with CaCC^43^, ^45^ and LRRC8 gene family products contribute to VRAC^46^, ^47^. The correlation between gene and functional channel identity is still somewhat unclear however, since there is also evidence to state that LRRC8 gene products are *non*-critical to VRAC function^48^. Our own recent qPCR studies of human cartilage show the presence of chloride channels CLCN-3, CLCN-7 and bestrophin, but we were not able to analyze either the anoctamin or LRRC8 families^49^. Here, we attempted to identify the underlying phenotype of *I*_Cl,vol_ using a pharmacological approach, since rabbit chloride channel gene sequences are not yet well characterized. There are no truly selective drugs for chloride channels, but DCPIB is considered somewhat selective for VRAC relative to other volume-activated chloride channel candidates such as CLC-3 and ANO-1^22^. AA is also thought to inhibit VRAC, but not ANO-1^23^. Conversely, CaCC_Inh_-A01 is thought selective for CaCC^21^ compared to VRAC. We chose optimal concentrations of agents, based on the literature quoted above and found both AA and DCPIB to powerfully inhibit control *I*_Cl,vol_. Interestingly, the apparently selective ANO-1 inhibitor CaCCInh-A01 also inhibited *I*_Cl,vol_ albeit significantly less potently than inhibitors of VRAC. Tentatively, our data suggests that both ANO-1- like and VRAC-like conductances may contribute to *I*_Cl,vol_. Comparison of Boltzmann parameters between control and ACLT chondrocytes shows a large increase in maximum conductance, but no change in slope (see Fig. 7). There is also, in this case, a shift of midpoint for voltage activation to the right (more positive potentials). This is surprising given the lack of shift in midpoint of the raw Boltzmann and supports the hypothesis that, following ACLT treatment, there is a substantial proportion of *I*_Cl,vol_ channel active under isotonic conditions, i.e., even before the application of hypotonic stretch. In all these scenarios, using an electrophysiological approach, it is not possible to determine if the profound increases in chloride conductance associated with ACLT result from the increased cell swell (shown in Fig. 2) or are independent of this. That there appears to be an increase of *I*_Cl,vol_ activity even under isotonic conditions following ACLT, may suggest the latter; increased functional expression. There are no direct proteomic or transcriptomic data for the rabbit ACLT OA model, but there is considerable evidence of changes in anion channel expression and DNA methylation in human studies, albeit of OA at advanced stages of progression. Specifically, recent microarray studies revealed significant changes in ANO-1/TEMEM16A^28^. Furthermore, LRRC8 genes also appear profoundly different in human OA. Next generation sequencing shows a 2.4 fold increase in LRRC8D (*adjusted p-val* 1.05e–6^50^) and fascinatingly, this is matched by a differentially methylation loci within LRRC8 genes^51^. Conversely, the hypo-osmotic state of more advanced degenerating cartilage itself may be expected to have an opposing effect on overall chloride conductance (i.e., decreasing chloride conductance), since *in vitro* studies, subjecting a human chondrocyte cell line to hypo-osmotic stress decreased expression of CLC-7/CLCN7^52^, a chloride channel especially involved with acid-base regulation.

In the light of these data, we hypothesize that increased activation of *I*_Cl,vol_ may occur at an early stage of OA and persist through its progression. Since volume-activated channels contribute to cell shrinkage, and cell shrinkage is a key component of apoptosis, this may be associated with the increase in caspase 3/7 activity. It should be noted, however, that we applied a hypo-osmotic solution for only a few minutes, whilst naturally, patients would have a decrease in joint fluid osmolality for many years. Future studies will be needed to determine if chloride channel inhibitor drugs or biologics are able to reduce progression of OA in pre-clinical models.

## Author contributions

(1) The conception and design of the study, or acquisition of data, or analysis and interpretation of data: KK FT CAS TM NO HM YM SI RBJ.

(2) Drafting the article or revising it critically for important intellectual content: KK FT CAS TM NO HM YM SI RBJ.

(3) Gave final approval of the version to be submitted: KK FT CAS TM NO HM YM SI RBJ.

## Conflict of interest

None of the authors has any conflict of interest in the outcomes of this study.

## Role of funding source

The funders had no role in the design, data collection and analysis, decision to publish, or the preparation of the manuscript.
