# Supplementary material for: Activation of a chondrocyte volume-sensitive Cl− conductance prior to macroscopic cartilage lesion formation in the rabbit knee anterior cruciate ligament transection osteoarthritis model
Source: Osteoarthritis Cartilage. 2016 Oct;24(10):1786–94. doi: 10.1016/j.joca.2016.05.019 (PMC5756537; doi:10.1016/j.joca.2016.05.019)
Supplement: Supplementary file 1 [file mmc1.docx]

**Supplementary Methods**

**Methods**

All experimental protocols conformed to The Guide for the Care and Use of Laboratory Animals (National Research Council 2011) and were approved by the Animal Care and Use Committee of Shiga University of Medical Sciences. All experiments used adult male white rabbits (body weight, 2.5 to 3kg).

Rabbits were deeply anaesthetized by intramuscular injection of ketamine (70mg/kg) and xylazine (5mg/kg). Right knees were shaved and disinfected with povidone-iodine solution. A medial parapatellar incision was made through the skin and an arthrotomy performed. The patella was dislocated laterally and the knee placed in full flexion. The ACL was visualized and transected. The joint was then irrigated with sterile saline and closed. For sham surgical controls, right knees were opened, the patella temporarily dislodged and the joint irrigated and closed. The ACL was not cut. Post-operatively, the animals were permitted cage activity without immobilization. The animals were closely monitored for health and welfare. At 2 or 4 weeks, both ACLT groups (5 rabbits each) were deeply anaesthetized (as above) and then humanely euthanized by intravenous injection of sodium pentobarbital (70mg/kg). At 4 weeks, the sham group (5 rabbits) was also euthanized, as described above.

***Histological examination:***

Dissected knees were fixed in 4% paraformaldehyde, decalcified in 4% EDTA solution, and embedded in paraffin blocks. 3μm thick sections were obtained from the femoral side of the patellofemoral joint and stained with toluidine blue and safranin-O as for proteoglycans and glycosaminoglycans. Randomly chosen histological sections were examined under the light microscope for histomorphometrical analysis using Image-Pro Plus (IPP) 6.0 software (Media Cybernetics Inc, Acton, MA). For overall evaluation of the cartilage area, tissues were graded by 3 blinded observers using both the Mankin score system^1^ and OARSI histopathology score^2^. Mankin scores identify ‘cartilage structure’, ‘cell distribution’, ‘safranin-O staining’ and ‘tidemark integrity’ as separate subitems. The sum of the separate scores range from 0 (normal) to 14 (severe OA). The OARSI system assesses the severity and the extent of cartilage surface involvement in the local osteoarthritic process. In contrast to the Mankin score and most other OA scores, the OARSI system emphasizes the extent of cartilage damage over the articular surface through a ‘stage’ component, in addition to damage, analyzed at several levels of the cartilage layer (i.e., ‘depth’ and ‘local cartilage damage’). ‘Grade’ (0 points for ‘normal’ up to six points for ‘severe’) and ‘Stage’ (0 points for ‘no OA activity seen’ up to four points for ‘>50% of articular surface affected’) can be used separately or can be combined in an overall score by multiplication.

***Isolation of rabbit articular chondrocytes:***

Articular chondrocytes were isolated using an enzymatic dissociation procedure similar to that described previously^3^ with modifications^4^. Articular cartilage was removed from bilateral knees and washed with phosphate-buffered saline. Sliced cartilage samples were incubated in plastic culture dishes containing Dulbecco’s modified Eagle’s medium (DMEM; Gibco, NY, USA) supplemented with 10% fetal calf serum and antibiotics in a humidified atmosphere of 95% air / 5% CO_2_ at 37°C for 1 to 3 days. On the day of the experiments, the cartilage samples were cut into small pieces (~1mm^3^) and digested with 0.5% collagenase (Type 2; Worthington Biochemical Corp., Lakewood, NJ, USA) for 4 h. Dispersed chondrocytes were washed three times, re-suspended in DMEM supplemented 40mM mannitol (~360mosmol/L) and used for experiments within 8h.

***Caspase-3/7 activity measurement:***

Caspase-3/7 activity was measured as an indicator of apoptosis. Briefly, cells were lysed and the supernatant collected for the measurement of caspase-3/7 activity using the Caspase-Glo 3/7 assay system (Promega, Madison, WI, USA) following the manufacturer’s instructions. The luminescent signal was measured with a luminometer (Infinite M200, Tecan, Männedorf, Switzerland).

***Cell swell assay:***

Chondrocytes suspensions were transferred to a recording chamber (0.5ml in volume) mounted on the stage of a Nikon TE2000-U microscope (Tokyo, Japan) and chondrocytes allowed to adhere to the glass bottom for at least 10min. The chamber was continuously perfused at a 2ml/min with an external solution at 36±1^o^C, and the external solution was exchanged by switching the perfusates at the inlet of the chamber, with a complete bath solution change taking 15-20s. Cell size measurements and patch-clamp experiments were conducted on round-shaped chondrocytes. Live chondrocytes microscopy images were captured (at 1 min intervals) before and during a hypo-osmotic challenge at a 2560×1920 pixel resolution using a CCD digital camera (DS-Fi1, Nikon) equipped with a DS-L2 control unit (Nikon). The cell cross-sectional areas were calculated using Image-J (NIH, Bethesda, MD, USA). These were each normalized to their respective initial iso-osmotic size. Swell data was fitted with differential equations simplified from Preston et al^5^ and Lewis et al^6^ using a custom MatLab script (MathWorks, MA, USA);

$${dvol}/{dt}=k\left( {osmo}_{in(t)}-{osmo}_{out} \right)$$

Equation 1

$${{dosmo}_{in}}/{dt}=G\left( {osmo}_{out}-{osmo}_{in(t)} \right)$$

Equation 2

where *k* and *G* are arbitrary constants for whole-cell water and osmolyte permeability respectively, *osmo_in(t)_* and *osmo_out_* are the intracellular and extracellular osmolalities. *vol* is the cell volume.

***Electrophysiology:***

Whole-cell membrane currents were recorded from isolated chondrocytes using an EPC-8 patch-clamp amplifier (HEKA, Lambrecht, Germany) or Axopatch 200 (Molecular Devices, USA). Fire-polished pipettes pulled from borosilicate glass capillaries (Narishige, Tokyo, Japan) had resistances of 2.0 to 4.0MΩ when filled. Square-step and voltage-ramp protocols were used to record whole-cell currents. Voltage ramps were used to monitor the time course of changes in membrane currents during various interventions, while the steady-state effects were recorded using square voltage steps, unless otherwise stated. Voltage-ramp protocols (*dV*/*dt* = ± 0.25*V/s*) were repeated every 6s and consisted of three phases: an initial +80mV depolarizing phase from a holding potential of –30mV, a second hyperpolarizing phase of –150mV and then a third phase returning to the holding potential. The current-voltage (*I-V*) relationship was measured during the second hyperpolarizing phase. Voltage-clamp protocols and data acquisition were controlled with Patchmaster software (HEKA, Lambrecht,Germany) or WinWCP (John Dempster, Sthrclyde University) and current records were filtered at 1kHz, digitized at 5kHz through an LIH-1600 interface (HEKA, Lambrecht, Germany), and stored on a Macintosh computer. Cell membrane capacitance (*C*_m_) was calculated from the capacitive transients elicited by 20ms voltage-clamp steps (±5mV) from a holding potential of –30mV, using the following relationship^7^: *C*_m_ = τ_c_ *I_0_*/*ΔV*_m_ (1-*I*_∞_/*I*_0_), where τ_c_ is the time constant of the capacitive transient, *I*_0_ is the initial peak current amplitude, *ΔV*_m_ is the amplitude of voltage step (5mV), and *I*_∞_ is the steady-state current value. The sampling rate for these measurements of *C*_m_ was 50kHz with a low-pass 10kHz filter. The average *C*_m_ for rabbit chondrocytes used in the present study was 7.2% (95% CI 6.39 - 8.03) pF (*n*=12, *N*=10). Membrane current amplitude and slope conductance were normalized to *C*_m_ in each cell and expressed as pA/pF, respectively. The zero current level is indicated by an arrowhead to the left of the current traces in the figures, all membrane potential values (*Vm*) have been corrected for junction potentials using JPCalc^8^.

*Difference currents*

Hypotonic-isotonic difference currents were calculated by subtracting individual currents under isotonic conditions, from the equivalents in hypotonic conditions.

*Conductance plots*

Boltzmann transformation of data separates the underlying whole cell ion conductance from the Ohmic driving force for ion flow. The procedure is to calculate the chord conductance for the current at each membrane potential; *g_(Vm)_* = I/(Vm-*V_rev_*) where *I* is the current, *Vm* the membrane potential, *V_rev_* is the current’s reversal potential and *g_(Vm)_* is the conductance at *Vm*. These data can then be fit by a Boltzmann equation^9^ as follows;

$$g_{(Vm)}=\frac{g}{(1+{\exp\left( V_{m}{-V}_{h} \right)}/k)}$$

Equation 3

where *g* is the maximal conductance, *V_h_* is the *Vm* at which the conductance is half-activated ( “midpoint”) and *k* is the slope of activation.

***Statistical analysis:***

Data are written as means (95% confidence intervals), with the number of animals (cell isolations) and cells from which measurements were made indicated by *N* and *n*, respectively. Statistical comparisons were made using either Student’s t-test or general linear model ANOVA (Minitab version 17, Minitab Ltd., Coventry UK) as stated. The caspase-3/7 data analysis was followed by a post-hoc Newman–Keuls test. The cell-swell ANOVA was followed with a post-hoc Dunnett’s test. Due to the large number of statistical tests surrounding the whole-cell Boltzmann transformed data and fits, we used Benjamini-Hochberg adjusted *p-values* assuming a 5% false detection rate^10^, calculated with R-studio (Boston, MA, USA). Differences were considered significant at *p*≤0.05. As per Journal instructions, exact *p-vals* are quoted, with categorization only when less than *p*≤0.0005.

***Solutions and Chemicals:***

The iso-osmotic external solution used for the patch-clamp experiments contained (in mM): mannitol 150, NaCl 100, MgCl_2_ 2.0, BaCl_2_ 2.0, GdCl_3_ 0.03, glucose 5.5, and Hepes 10 (pH adjusted to 7.4 with NaOH). The osmolality of these external solutions, measured with a freezing point depression osmometer (Fiske, Burlington, MA, USA), averages 360 mOsm. This osmolality was chosen because it resembles that of native cartilage. In addition, the volume sensitive response in bovine chondrocytes is attenuated at the osmolality commonly used for other cell types (~280 mOsm)^11^. The standard pipette solution contained (in mM): caesium aspartate 135, CsCl 30, tetraethylammonium chloride 20, MgCl_2_ 2.0, Tris-ATP 5.0, Na_2_-GTP 0.1, EGTA 5.0, and Hepes 5.0 (pH adjusted to 7.2 with CsOH). The concentrations of free Ca^2+^ and Mg^2+^ in the pipette solution were calculated to be approximately; *pCa^2+^*=9.8 and *pMg^2+^*=4.3 ^11, 12^. The iso-osmotic external solution used for measuring cell swell contained (in mM): mannitol 180, NaCl 90, KCl 5.4, CaCl_2_ 1.8, MgCl_2_ 0.5, NaH_2_PO_4_ 0.33, glucose 5.5, and Hepes 5.0 (pH adjusted to 7.4 with NaOH). The hypo-osmotic external solution was made by omitting mannitol. DCPIB and arachidonic acid were purchased from Sigma-Aldrich (Japan and UK, offices), but CaCCinh-A01 was purchased from Tocris Bioscience (Bristol, UK). These were initially dissolved in DMSO and diluted to final concentrations on the day of experiments. DMSO was added to the matching control flow solutions as appropriate.

1 Mankin HJ, Dorfman H, Lippiello L, Zarins A. Biochemical and metabolic abnormalities in articular cartilage from osteo-arthritic human hips. II. Correlation of morphology with biochemical and metabolic data. J Bone Joint Surg Am 1971; 53: 523-537.

2 Pritzker KP, Gay S, Jimenez SA, Ostergaard K, Pelletier JP, Revell PA, et al. Osteoarthritis cartilage histopathology: grading and staging. Osteoarthritis Cartilage 2006; 14: 13-29.

3 Wilson JR, Duncan NA, Giles WR, Clark RB. A voltage-dependent K+ current contributes to membrane potential of acutely isolated canine articular chondrocytes. J Physiol 2004; 557: 93-104.

4 Okumura N, Imai S, Toyoda F, Isoya E, Kumagai K, Matsuura H, et al. Regulatory role of tyrosine phosphorylation in the swelling-activated chloride current in isolated rabbit articular chondrocytes. J Physiol 2009; 587: 3761-3776.

5 Preston GM, Carroll TP, Guggino WB, Agre P. Appearance of water channels in Xenopus oocytes expressing red cell CHIP28 protein. Science 1992; 256: 385-387.

6 Lewis R, Asplin KE, Bruce G, Dart C, Mobasheri A, Barrett-Jolley R. The role of the membrane potential in chondrocyte volume regulation. J Cell Physiol 2011; 226: 2979-2986.

7 Benitah JP, Gomez AM, Bailly P, Da Ponte JP, Berson G, Delgado C, et al. Heterogeneity of the early outward current in ventricular cells isolated from normal and hypertrophied rat hearts. J Physiol 1993; 469: 111-138.

8 Barry PH. JPCalc, a software package for calculating liquid junction potential corrections in patch-clamp, intracellular, epithelial and bilayer measurements and for correcting junction potential measurements. J Neurosci Methods 1994; 51: 107-116.

9 Barrett-Jolley R, Pyner S, Coote JH. Measurement of voltage-gated potassium currents in identified spinally-projecting sympathetic neurones of the paraventricular nucleus. Journal of Neuroscience Methods 2000; 102: 25-33.

10 Benjamini Y, Hochberg Y. Controlling the False Discovery Rate - a Practical and Powerful Approach to Multiple Testing. Journal of the Royal Statistical Society Series B-Methodological 1995; 57: 289-300.

11 Hall AC. Volume-sensitive taurine transport in bovine articular chondrocytes. J Physiol 1995; 484 ( Pt 3): 755-766.

12 Tsien RY, Rink TJ. Neutral carrier ion-selective microelectrodes for measurement of intracellular free calcium. Biochim Biophys Acta 1980; 599: 623-638.
